# Drug Distribution After Intravitreal Injection: A Mathematical Model

**DOI:** 10.1167/iovs.65.4.9

**Published:** 2024-04-03

**Authors:** Alessia Ruffini, Alessia Casalucci, Caterina Cara, C. Ross Ethier, Rodolfo Repetto

**Affiliations:** 1 Department of Civil, Chemical and Environmental Engineering, University of Genoa, Genoa, Italy; 2 Department of Civil, Environmental and Architectural Engineering, University of Padua, Padua, Italy; 3 Wallace H. Coulter Department of Biomedical Engineering, Georgia Institute of Technology/Emory University, Atlanta, GA, United States

**Keywords:** intravitreal drug delivery, eye deflation, mathematical modeling, age-related macular degeneration

## Abstract

**Purpose:**

Intravitreal injection of drugs is commonly used for treatment of chorioretinal ocular pathologies, such as age-related macular degeneration. Injection causes a transient increase in the intraocular volume and, consequently, of the intraocular pressure (IOP). The aim of this work is to investigate how intravitreal flow patterns generated during the post-injection eye deflation influence the transport and distribution of the injected drug.

**Methods:**

We present mathematical and computational models of fluid motion and mass transport in the vitreous chamber during the transient phase after injection, including the previously unexplored effects of globe deflation as ocular volume decreases.

**Results:**

During eye globe deflation, significant fluid velocities are generated within the vitreous chamber, which can possibly contribute to drug transport. Pressure variations within the eye globe are small compared to IOP.

**Conclusions:**

Even if significant fluid velocities are generated in the vitreous chamber after drug injection, these are found to have negligible overall effect on drug distribution.

Intravitreal drug injections are routinely used to deliver drugs for the treatment of certain types of chorioretinal disease, most commonly for delivery of intravitreal anti-VEGF agents for the treatment of neovascular age-related macular degeneration but also to treat diabetic macular edema, retinal vein occlusion, and non-infectious uveitis.^1^ Such injections are now very widely used; for example, one review, drawing from a single US database, identified more than 800,000 intravitreal injections being administered over an 18 month period to treat neovascular age-related macular degeneration.^2^

When a drug is injected into the vitreous chamber, it can be transported by diffusion and by advection, the latter mechanism being related to a very slow fluid motion from the anterior segment of the eye to the retina, due to a combination of aqueous production at the ciliary processes and active fluid pumping by the retinal pigment epithelium (RPE). Transport processes in the vitreous chamber have been studied in a number of works that have employed numerical analyses,^3^^–^^7^ in vitro experiments,^8^^,^^9^ and in vivo measurements.^10^ Various authors^3^^–^^5^ have used numerical simulations to show that, even if the velocities associated with this bulk percolation flow are very small ($∼10^{-8}$ m/s),^11^ advection is an important transport mechanism, especially in the case of large molecules with small diffusivities. Further, various authors have shown that vitreous motion induced by eye rotations can also play a key role in transport processes in the vitreous chamber.^9^^,^^12^^–^^14^

In the case of drug injection into brain tissue, Basser^15^ showed with a mathematical model that fluid flow can also be generated by another mechanism. The tissue is locally pressurized at the injection site, which drives fluid away. This fluid motion can be explained making use of Biot’s consolidation model, which is a classic theory in soil mechanics.^16^ Basser’s work for transport in the brain is based on the assumption that the brain tissue is a porous material of infinite extent. Chan et al.^17^ have speculated that a similar mechanism can take place in the eye after a vitreoretinal injection, even if Basser’s theory cannot be directly applied in this case as the eye has finite size and an intraocular injection will lead to scleral expansion and a pressure elevation.

The injection of a drug-containing fluid into the eye, typically 50 µL delivered via a needle, leads to a well-documented transient increase in IOP,^18^^–^^20^ which is related to the compliance of the ocular globe.^21^^,^^22^ This IOP increase resolves as fluid drains from the eye at a greater than normal rate. Although several studies have considered intraocular flow patterns associated with an intravitreal injection,^23^^–^^25^ they have not included the effects of transient globe expansion and deflation. In this work, we present a mathematical model to study fluid motion and mass transport within the vitreous chamber after an intravitreal drug injection. Our aim is to assess whether and to what extent this flow may contribute to transporting the injected drug within the vitreous chamber. The problem is of clinical relevance since understanding drug transport processes within the vitreous body is key to predicting the fate of the drug in the eye, particularly the regions of the retina that will preferentially receive the treatment and the associated time scales.

## Mathematical Model

We model the eye as an elastic sphere filled with vitreous humor, which is assumed to be an incompressible, poroelastic medium. Although the vitreous does not entirely fill the globe, this is a reasonable approximation, since the vitreous volume is a major part of total globe volume, the other components are effectively incompressible, and (as will be seen below) the vast majority of the relevant fluid mechanical effects occur in the vitreous. The injected fluid is modeled as Newtonian and incompressible. Given an injected volume of *V_inj_* = 50 µL and an injection flow rate of *Q_inj_* = 1 mL/min,^26^ we can estimate the injection time as *t_inj_* = *V_inj_*/*Q_inj_* ≈ 3 s. This is very rapid compared to the subsequent dynamics (verified a posteriori); hence, we here simply model eye globe deflation, assuming that the injection is effectively instantaneous. We therefore take time zero to be immediately after the injection, when an increase in ocular volume (equal to the injection volume) has produced a pressure increase, which, in turn, drives flow out of the eye during deflation.

### Zero-Dimensional Model of Eye Globe Deflation

It is convenient to review the well-established formulation of a zero-dimensional transient model to describe deflation of the eye globe, which leads to a problem governed by a simple time-dependent ordinary differential equation (ODE). Mass conservation requires that variation over time of the eye volume is related to the difference between fluid flux into the eye and out of it. This is mathematically expressed as

(1) $$\begin{array}{c} \frac{dV_{o}}{dt}=Q_{in}-Q_{out},\; \end{array}$$

with *t* time, *V_o_* = *V_o_*(*t*) the total volume of the eye, *Q_in_* the rate of aqueous humor production by the ciliary processes minus rate of pressure-independent outflow, and *Q_out_* the rate of pressure-dependent outflow. We assume that *Q_in_* is constant and equal to 2.2 µL/min.^27^

IOP (denoted by *P*) is related to globe volume *V_o_* through ocular compliance, and *C* = *dV_o_*/*dP*, which is assumed to be constant and equal to 1 µL*/*mm Hg.^21^ This quantifies the capacity of the eye to change its volume in response to an IOP change. Moreover, we describe pressure-dependent outflow by

(2) $$\begin{array}{c} P-P_{epv}=Q_{out}R,\; \end{array}$$

where *P_epv_* is the episcleral venous pressure, and R is the hydrodynamic resistance to fluid drainage, assumed to be constant. Episcleral venous pressure depends on posture and other factors; we here assume *P_epv_*= 8 mm Hg.^28^^–^^30^

Under physiologic conditions (indicated hereafter with a hat over the corresponding variable), we have $Q_{in}={\hat{Q}}_{out},$ from which we obtain$\;R=\left(\hat{P}-P_{epv}\right)/Q_{in}$. In other words, the resistance can be computed as the difference between the physiologic IOP and episcleral vein pressure, divided by the aqueous production rate. Substituting the above expressions into (1), we obtain

(3) $$\begin{array}{ccc} \frac{dP}{dt} & = & \frac{1}{C}\left(Q_{in}-\frac{P-P_{epv}}{R}\right)\\ & = & \frac{1}{C}\left(Q_{in}-\frac{P-\hat{P}+\hat{P}-P_{epv}}{R}\right)=-\frac{P-\hat{P}}{RC},\; \end{array}$$

where we have used the fact that $Q_{in}={\hat{Q}}_{out}=\left(\hat{P}-P_{epv}\right)/R$. This equation shows that the variation in time of IOP is proportional to the difference between the actual value of IOP and the physiologic one and inversely proportional to ocular compliance and resistance to aqueous drainage.

The ODE (3) can be solved subject to an initial condition that specifies IOP at the initial time, *P*(0), which in turn depends on the volume of the injected fluid *V_inj_* through the expression $P\left(0\right)=V_{inj}/C+\hat{P}$. The resulting analytical solution is

(4) $$\begin{array}{c} P=\hat{P}+\frac{V_{inj}}{C}\exp\left(-\frac{t}{RC}\right),\;V_{o}=\hat{V}+V_{inj}\exp\left(-\frac{t}{RC}\right).\; \end{array}$$

The above relationships show that both pressure and volume decrease exponentially in time after injection, with time scale τ=RC.

### Three-Dimensional Model of Eye Globe Deflation

#### Formulation of the Problem

**Figure 1.**
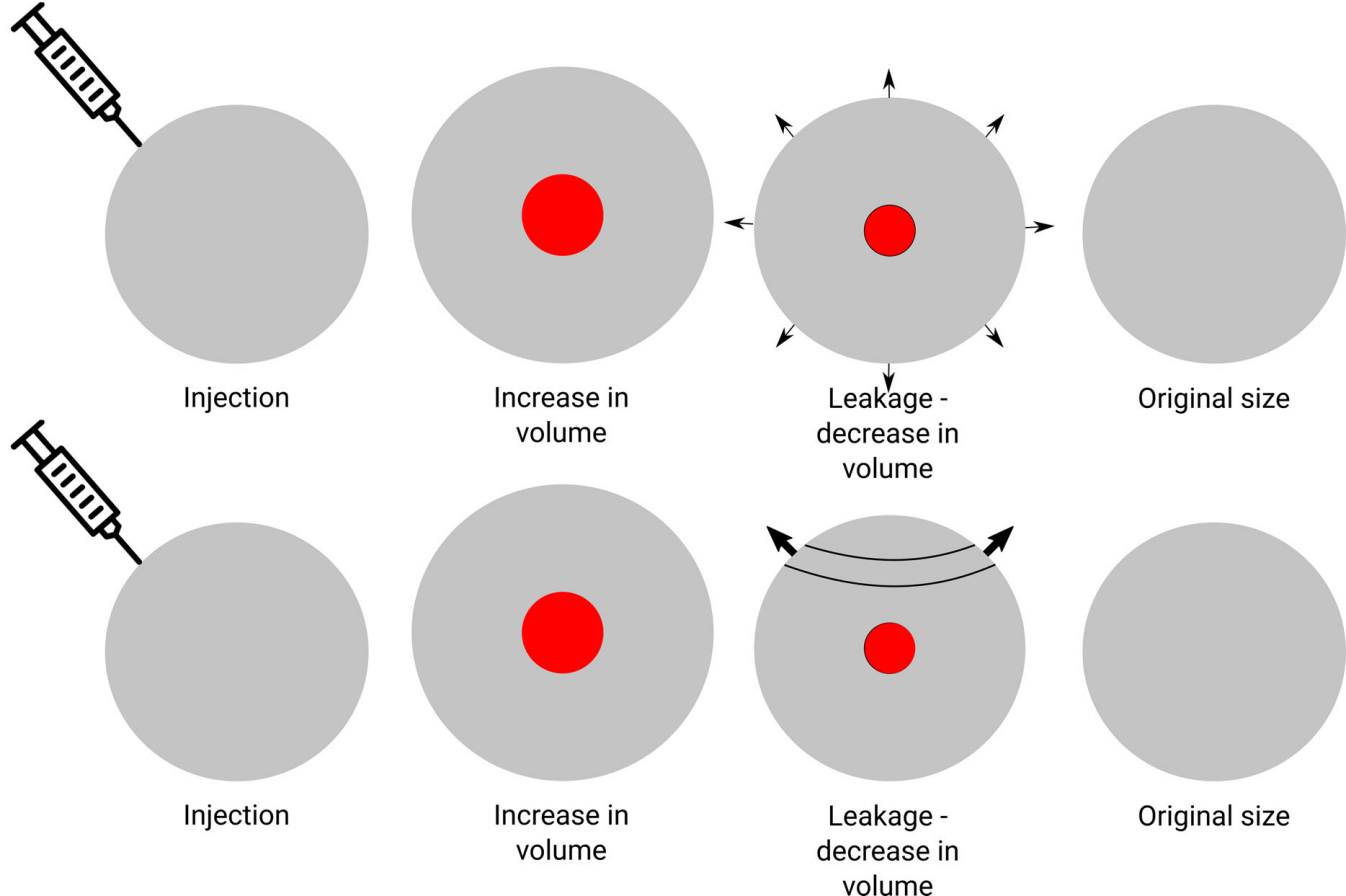
Sketch of the four different phases during intravitreal injection treatment for case A (*top*) and case B (*bottom*). The *red circle* represents the injected bolus of drug-containing fluid. *Arrows* represent the drainage of fluid out of the eye.

We now investigate the flow generated in the vitreous chamber during the deflation phase and its effect on drug transport. We idealize the problem by assuming that the drug solution is injected into the center of the vitreous and expands uniformly during injection, so that a spherical “bubble” of drug solution forms, whose center coincides with the center of the eye. This is schematically illustrated in Figure 1, where, in each row, the second sketch depicts the initial time, with an increased eye volume and the liquid pool containing the drug in the center. We further assume that, at all times, the geometry of the domain can be described as two concentric spheres with time-varying radii: the outer sphere (with volume *V_o_*) represents the eye globe, while the inner sphere (with volume *V_i_*) represents the bolus of injected fluid (see Fig. 1). The space between the two spheres is occupied by the vitreous humor, whereas the inner sphere contains only the injected fluid (including the drug). We assume that the injected fluid displaces the vitreous body, creating an inner fluid bolus, without changing vitreous porosity. Thus, at the initial time, the volume of the inner sphere coincides with the injected volume, $V_{i}\left(0\right)=V_{inj}=V_{o}\left(0\right)-\hat{V}$.

The contribution of stresses within the sclera in supporting the increased pressure in the eye, secondary to the injection, is expected to be much greater than the contribution due to stresses in the poroelastic vitreous body. In brief, the elastic modulus of the sclera is approximately five orders of magnitude larger than that of the vitreous.^31^^,^^32^ We use this fact, as well as the formula for the expansion of a thick-walled internally pressurized elastic incompressible sphere, to compute the relative expansion expected from 1) the vitreous alone containing an internally pressured bolus of fluid and 2) the scleral shell alone, which is acted upon internally by the IOP. Specifically, if the sphere has external and internal undeformed radii of *a* and *b*, respectively, then $\Delta a=\frac{3\hat{P}a}{4E}\left(\frac{1}{{\left(a/b\right)}^{3}-1}\right)$, where $\hat{P}$ is the internal pressure (IOP in physiologic conditions), *E* is the Young's modulus of the sphere wall, and Δ*a* is the expansion of the outer surface of the sphere.^33^ Using superscripts *S* and *V* to denote sclera alone and vitreous, the internal radius for the vitreous is that of the injected fluid bolus (*b^V^* ≈ 2.3 mm) and the external radius is approximately the radius of the eye (*a^V^* ≈ 1.1 cm for a human eye). In the case of the sclera, the internal radius is approximately *b^S^* = *a^V^*, while the external radius *a^S^* = *b^S^* + *h*, where *h* is the scleral thickness (≈ 670 µm). Noting that (*a^V^*/*b^V^*)^3^ ≫ 1 and *h*/*b^S^* ≪ 1, the ratio of the expansion of the sclera alone to the vitreous alone can be expressed as

(5) $$\begin{array}{c} \frac{\Delta a^{S}}{\Delta a^{V}}\approx \frac{E^{V}}{E^{S}}\frac{\hat{R}}{3h}\frac{\hat{V}}{V_{inj}},\; \end{array}$$

where $\hat{R}$ (≈ *a^V^*) is the physiologic radius of the eye. This ratio is approximately ( ≈ 10^−5^)( ≈ 5)( ≈ 120) ≈ 0.006 ≪ 1. This shows that the contribution of the vitreous in withstanding the internal pressure is negligible.

The vitreous also has a viscosity, which produces viscous stresses during the deflation phase. However, this contribution is also negligible when compared to the resistance to fluid outflow from the eye globe. This is demonstrated by the fact that the typical relaxation time of the vitreous body is less than 1 second (estimate based on data reported in Tram et al.^31^) and thus much shorter than the deflation time of the eye of interest in our model, which is of tens of minutes.

For the above reasons, we do not solve an equilibrium equation for the vitreous and simply assume that it behaves as an incompressible material. Given the above assumption, the variation in time of the outer and inner volumes are equal, so that *dV_o_*/*dt* = *dV_i_*/*dt*. The radii of the two spheres are computed as

(6) $$\begin{array}{c} R_{o}\left(t\right)=\sqrt[{3}]{\frac{3V_{o}\left(t\right)}{4\;\pi }},\;R_{i}\left(t\right)=\sqrt[{3}]{\frac{3\left(V_{o}\left(t\right)-\hat{V}\right)}{4\;\pi }},\; \end{array}$$

where *V_o_*(*t*) is given by Equation (4). We note that, since we assume that both the injected fluid and the vitreous body are incompressible materials, the increase in pressure within the domain during injection is uniform and instantaneous.

Owing to the spherical symmetry of the domain and of vitreous displacement field, the incompressibility condition imposes

(7) $$\begin{array}{c} {\dot{u}}_{r}=\frac{{\dot{V}}_{o}\left(t\right)}{4\pi r^{2}},\; \end{array}$$

with *r* denoting the radial coordinate, *u_r_* denoting the radial component of vitreous displacement, and over dots indicating differentiation with respect to time.

Fluid percolates through the vitreous body, and according to Darcy's law for fluid flow in a porous medium, the following relationship holds:

(8) $$\begin{array}{c} q\;=v-\dot{u}=-\frac{k}{\mu }\nabla p,\; \end{array}$$

where ***v*** is fluid velocity in the fixed frame, ***u*** is tissue displacement, *k* is vitreous permeability to water, μ is the dynamic viscosity of the percolating fluid, and *p*(***x***, *t*) is the pressure, which is now a function of both time and position. Note that the above equation accounts for the fact that also the solid matrix of the tissue can possibly move. To distinguish the pressure used in Darcy's law from the IOP in the zero-dimensional model, we here denote pressure with a small *p*, as opposed to the capital *P* used in the previous section. Taking the divergence of the above expression, assuming that both fluid and solid matrix are incompressible and that *k* does not vary with space, we find that the pressure satisfies Laplace's equation, which is a statement of mass conservation, and reads

(9) $$\begin{array}{c} \nabla^{2}p=0.\; \end{array}$$

Solute (drug) transport is governed by the advection diffusion equation that can be written as

(10) $$\begin{array}{c} \frac{\partial c}{\partial t}=\nabla \cdot \left(D\nabla c\right)-\nabla \cdot \left(vc\right),\;\; \end{array}$$

where *c* is the concentration of injected drug and *D* is the diffusion coefficient (assumed constant).

**Figure 2.**
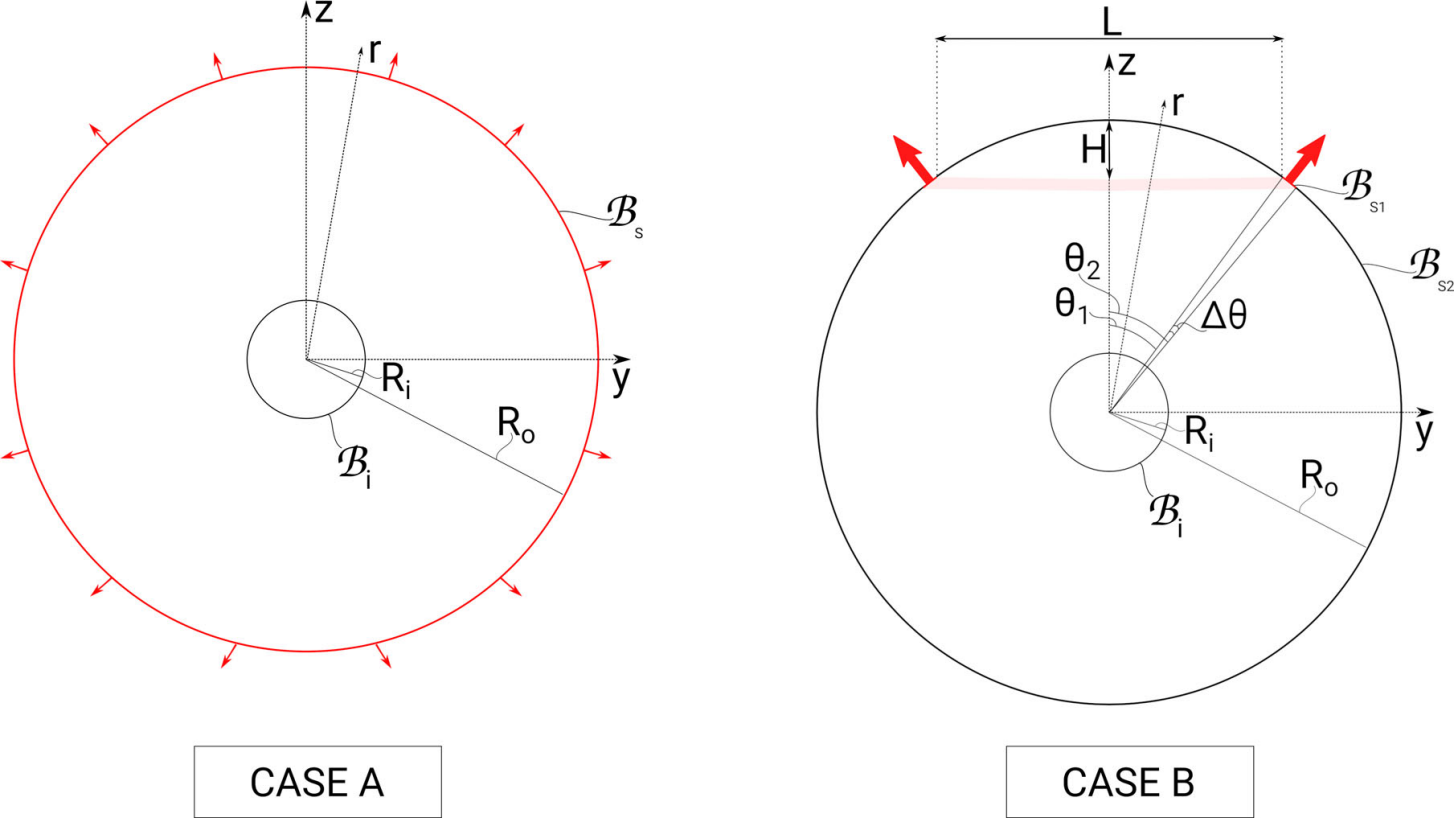
Sketch of the domain for cases A and B, consisting of an inner fluid bolus (*black circle*) centered within the eye globe (*red circle* in case A, *red/black circle* in case B). *Red outward-pointing arrows* signify fluid exiting the eye globe. $B_{i}\;$ is the inner fluid bolus surface (radius *R_i_*), and $B_{s}$ is the scleral surface (radius *R_o_*). In case B, $B_{s1}$ is the surface from which fluid exits the domain, and $B_{s2}$ is the rest of the eye globe surface.

We solve Equations (9) and (10) in a domain bounded by the two spherical surfaces (see Fig. 2), the radii of which change over time, according to Equation (6). The initial condition imposes that the pressure is constant in the domain and equal to *P*(0).

Equations (9) and (10) require boundary conditions. We consider the following two limiting cases.

• *Case A.* Fluid outflow is assumed to be spherically symmetric and to thus involve the whole corneoscleral surface, as shown in Figure 1, top row, and Figure 2A.
• *Case B*. Fluid outflow occurs entirely through the trabecular meshwork and is thus localized to a specific region of the corneoscleral surface, as depicted in Figure 1, bottom row, and Figure 2B.

*Case A:* At the scleral surface ($B_{s}$), we impose a constant water flux

(11) $$\begin{array}{c} q\cdot {\left.n\right|}_{B_{s}}=q_{r}=-\frac{k}{\mu }\frac{dp}{dr}=-\frac{{\dot{V}}_{o}}{4\pi R_{o}^{2}},\; \end{array}$$

where ***n***  is the outward unit normal vector to the scleral surface, which is purely radial. The value of the fluid flux imposed (right hand side of Equation (11)) is derived from the zero-dimensional solution of the Zero-Dimensional Model of Eye Globe Deflation section.

Since the problem has spherical geometry, fluid velocity is purely radial and the incompressibility condition implies that *v_r_* is proportional to *r*^−2^, similar to the dependence of vitreous displacement on *r*. Equation (7) and condition (11) immediately show that

(12) $$\begin{array}{c} v_{r}=0.\; \end{array}$$

Since fluid velocity in the laboratory frame is zero, there is no advective contribution to solute transport and Equation (10) reduces to the diffusion equation. In this case, the concentration is just function of *r* and *t*, and this equation reads

(13) $$\begin{array}{c} \frac{\partial c}{\partial t}=D\frac{1}{r}\frac{\partial }{\partial r}\left(r\frac{\partial c}{\partial r}\right).\; \end{array}$$

We assume that solute cannot cross the scleral surface; thus, at the scleral boundary, we impose a zero solute flux condition

(14) $$\begin{array}{c} {\left.q_{s}\cdot n\right|}_{B_{s}}=0.\; \end{array}$$

Solute flux takes the general form ***q**_s_* = −*D*∇*c* + *c**v***, and in this case, ***v***  =  **0**. Thus, the above equation reads $-D\frac{dc}{dr}=0$ on $\;B_{s}.$ Condition (14) could be replaced by an adsorption condition at the retina. However, over the time scale of our simulations, the concentration of the drug at the boundary is negligible, which means that condition (14) is effectively correct.

At the surface of the inner fluid bolus ($B_{i}$), we impose the values of the pressure and drug concentration (Dirichlet boundary conditions)

(15) $$\begin{array}{c} {\left.p\right|}_{B_{i}}=P\left(t\right),\;\;\;\;\;\;\;\;\;\;{\left.c\right|}_{B_{i}}=c_{0},\; \end{array}$$

where the imposed pressure *P*(*t*) is determined from Equation (4), under the assumption that the pressure drop across the vitreous is small compared to the IOP (justified a posteriori). Moreover, *c*_0_ is the drug concentration in the injected fluid.

*Case B:* Here the scleral surface is subdivided into two regions $B_{s}=B_{s1}\cup B_{s2}$, where $B_{s1}$ is the surface from which fluid exits the domain (the trabecular meshwork region) and $B_{s2}$ is the rest of the eye globe surface now considered impermeable, as shown in Figure 2 (case B). Using spherical coordinates, $B_{s1}$ is the region defined by θ_1_ ≤ θ ≤ θ_2_ and 0 ≤ ϕ < 2π, with θ and ϕ being the polar and azimuthal angular coordinates, respectively. We impose

(16) $$\begin{array}{c} q\cdot {\left.n\right|}_{B_{s1}}=\frac{Q}{A_{s1}}=-\frac{{\dot{V}}_{o}}{A_{s1}},\; \end{array}$$

(17) $$\begin{array}{c} q\cdot {\left.n\right|}_{B_{s2}}=0,\; \end{array}$$

where *A*_*s*1_ is the area of the outlet region $B_{s1}$, which is equal to $2\pi R_{o}^{2}\left(\cos\theta_{2}-\cos\theta_{1}\right)$. For modeling solute transport, we impose zero flux across the sclera on $B_{s2}$, as for case A, and zero diffusive flux on $B_{s1}$.

The conditions at the inner boundary ($B_{i}$) are the same as for case A and are given in Equation (15).

#### Solution

Equations (9) and (10) have been implemented in weak form in COMSOL Multiphysics and solved numerically, assuming axial symmetry. Deformation of the domain was modeled using the “deformed geometry” tool. The typical mesh used in the simulations consisted of approximately 15,000 unstructured triangular elements, with a corresponding mean elemental density of 3.9 × 10^7^ elements/m^2^. We conducted mesh invariance tests using eight meshes, increasing the number of elements by ∼10% in each mesh and sampling the computed pressure at five fixed locations. The mesh used for production runs showed a variation of pressure of less than 1% with respect to the most refined one.

An analytical solution for fluid motion, which is governed by the Laplace Equation (9), can also be found, taking advantage of the spherical shape of the domain and assuming that the outflow region is so small that it can be modeled as a line sink (equivalent to a point sink in our axisymmetric model). In the limit of θ_2_ → θ_1_, the condition (16) reads

(18) $$\begin{array}{c} {\left.-\frac{k}{\mu }\frac{\partial p}{\partial r}\right|}_{r=R_{o}\left(t\right)}=\frac{Q\left(t\right)\delta \left(\theta -\theta_{1}\right)}{2\;\pi \;\sin\theta_{1}\;R_{o}^{2}\left(t\right)},\; \end{array}$$

where δ(θ − θ_1_) denotes the Dirac delta function, centered at θ_1_, and $Q\left(t\right)=-{\dot{V}}_{o}\left(t\right)$ is the fluid outflow rate from the globe. The above condition satisfies the requirement that the total outflow rate is *Q*(*t*). At the inner spherical surface, *r* = *R_i_*, we still use the Dirichlet condition on the pressure (15).

We use separation of variables and find that the solution to the Laplace equation can be written in terms of an infinite sum of Legendre polynomials,^34^ in the form

(19) $$\begin{array}{c} p\left(r,\theta \right)=\sum_{l=0}^{\infty }\alpha_{l}r^{l}P_{l}\left(cos\theta \right)+\sum_{l=0}^{\infty }\beta_{l}\frac{1}{r^{l+1}}P_{l}\left(cos\theta \right),\; \end{array}$$

where *P_l_* is the Legendre polynomial of degree *l* and we have not included the modified Legendre polynomials, since they are singular at the poles, which is not physical in our case. The coefficients α_*l*_ and β_*l*_ can be determined by imposing the boundary conditions at *R_i_* and *R_o_* and taking advantage of the orthogonality properties of the Legendre polynomials to obtain

(20) $$\begin{array}{ccc} \alpha_{l} & = & -\frac{1}{R_{i}^{2l+1}}\frac{Q}{4k\pi R_{o}^{2}}P_{l}\left(cos\theta_{0}\right)\frac{2l+1}{l\frac{R_{o}^{l-1}}{R_{i}^{2l+1}}+\frac{l+1}{R_{o}^{l+2}}},\\ \beta_{l} & = & \frac{Q}{4k\pi R_{o}^{2}}P_{l}\left(cos\theta_{0}\right)\frac{2l+1}{l\frac{R_{o}^{l-1}}{R_{i}^{2l+1}}+\frac{l+1}{R_{o}^{l+2}}}.\; \end{array}$$

This analytical solution matches very closely the numerical results presented in the Results section (not shown).

### Parameter Values

The angle θ_1_ shown in Figure 2 has been estimated using the maximum depth *H* and maximum diameter *L* of the anterior chamber and the radius of the eye ${\hat{R}}_{o}$ (Fig. 2), according to the following relationship:

(21) $$\begin{array}{c} \theta_{1}=\tan^{-1}\frac{L/2}{{\hat{R}}_{o}-H}.\; \end{array}$$

Assuming *H* = 2.63 mm,^35^ *L* = 13 mm,^36^ and ${\hat{R}}_{o}=11.3\;\;$mm, the above equation gives θ_1_ = 39°.

The area subtended by aqueous drainage structures (i.e., the area of the trabecular meshwork) can be expressed as

(22) $$\begin{array}{c} A_{s1}=\int_{0}^{2\pi }\int_{\theta_{1}}^{\theta_{2}}{{\hat{R}}_{o}}^{2}\sin\theta \;d\theta \;d\phi =2\pi {{\hat{R}}_{o}}^{2}\left(\cos\theta_{1}-\cos\theta_{2}\right).\; \end{array}$$

Following Heys et al.,^37^ we assume a value of *A*_*s*1_ = 18 mm^2^. Using this value and (22), we find Δθ = θ_2_ − θ_1_ ≈ 2°.

The aqueous outflow resistance R of the pressure-dependent outflow pathway is estimated as explained in the Zero-Dimensional Model of Eye Globe Deflation section, according to the following equation:

(23) $$\begin{array}{c} R=\frac{\left(\hat{P}-P_{epv}\right)}{Q_{out}}.\; \end{array}$$

We use as physiologic IOP the value $\hat{P}=15$ mm Hg and an episcleral venous pressure of *P*_*epv*_ = 8 mm Hg.^28^^–^^30^ The aqueous humor flow rate minus the pressure insensitive outflow rate is taken equal to 2.2 µL/min.^38^^,^^39^ Based on these values, we obtain R=3.18mm Hg min µL^−1^. In Goel's work,^40^ an estimated value for the resistance of the conventional aqueous drainage tissues ranges between 3 and 4 mm Hg min µL^−1^, which is consistent with our choice.

We focus here on the injection and subsequent transport of bevacizumab, a commonly injected drug. We estimated bevacizumab's diffusion coefficient in the vitreous by first using the Stokes–Einstein equation to estimate the diffusivity in free solution (unhindered diffusion):

(24) $$\begin{array}{c} D=\frac{k_{B}T}{6\pi \mu a},\; \end{array}$$

where *k_B_* is Boltzmann's constant (1.38 × 10^−23^ J/K), *T* is the absolute temperature (310 K), and *a* denotes the hydrodynamic radius of the diffusing species. For bevacizumab, we have *a* = 4.58 × 10^−9^ m,^41^ which yields *D* = 7 × 10^−11^ m^2^s^−1^, in line with the value derived by Hutton-Smith et al.^42^ The question then arises as to the appropriate value of *D* in the vitreous, where some degree of diffusive hindering may occur. The major components of the vitreous humor are collagen and hyaluronan, and we here estimate the pore sizes in the vitreous from knowledge of their concentrations. We first consider collagen, whose concentration in the bovine vitreous is 1.1  × 10^−2^ weight %,^43^ which corresponds to a collagen solid fraction of ϕ = 8 × 10^−5^, considering a density of collagen fiber of ρ_*c*_ = 1420  kg/m^3^.^44^ Idealizing collagen fibrils in the vitreous as uniformly distributed cylinders of radius *a_c_* = 12.5 nm,^45^ the solid fraction can be expressed as $\phi =\pi {\left(\frac{a_{c}}{b}\right)}^{2}$, where *b* is the side of a “squared unit cell” around each collagen fiber,^46^ which is equal to half the characteristic interfiber spacing. Based on this approach, we compute a characteristic interfiber spacing of 2.5 × 10^−6^ m, which is far larger than the hydrodynamic radius of bevacizumab, from which we conclude that collagen in the vitreous does not appreciably hinder the diffusion of bevacizumab. A similar calculation can be repeated for hyaluronan, using a concentration of 2 × 10^−2^ weight % (ϕ =    6.1 × 10^−5^, with hyaluronan density ρ_*h*_ ≈ 1800 kg/m^3^) and fiber radius of 0.5 nm, yielding a characteristic interfiber spacing of 1.1 × 10^−7^ m, which again is much larger than the hydrodynamic radius of bevacizumab. We therefore expect that, to a first approximation, diffusive hindrance due to the vitreous is modest, and we therefore use the free solution value for the diffusion coefficient of bevacizumab in our calculations, namely, *D* = 7 × 10^−11^ m^2^s^−1^. All the parameters used in this work are summarized in Table.

## Results

**Figure 3.**
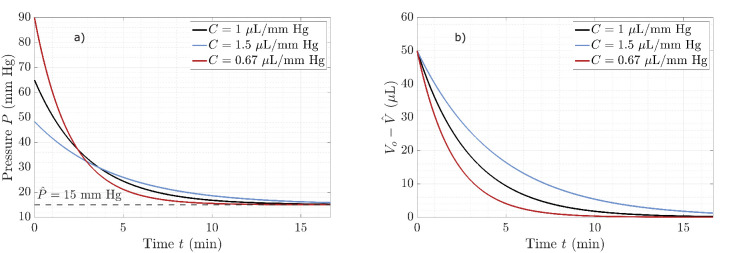
Variation of intraocular pressure P (**a**) and volume V (**b**) versus time for three values of ocular compliance, *C*. The black curves correspond to the baseline ocular compliance value (*C* = 1 µL/mm Hg), while the *blue and red curves* correspond to ocular compliance values 1.5 times larger and smaller than the baseline value, respectively. The dashed line in (**a**) represents the physiologic (equilibrium) value of intraocular pressure, while $\hat{V}$ is the equilibrium physiologic intraocular volume.

Figure 3 shows the variation of IOP and the “excess” ocular volume $\left(V_{o}\left(t\right)-\hat{V}\right)$ with time, for three different values of ocular compliance. We observe the expected inverse relationship between compliance and initial pressure, with relaxation time scales varying with the ocular compliance. The maximum value of the radial velocity of the eye wall occurs at the initial time and, using the parameters of Figure 3 (black curve), is approximately 0.2 µm/s.

In case A, the fluid is stationary in the laboratory frame (see Methods). This somewhat counterintuitive result means that fluid flux relative to the vitreous body is effectively produced by the shrinking of the domain, that is, the deflation of the globe makes the vitreous to move inward toward the center of the globe, causing the stationary fluid to percolate through the porous vitreous. This theoretical prediction has been validated numerically using COMSOL Multiphysics (data not shown).

**Figure 4.**
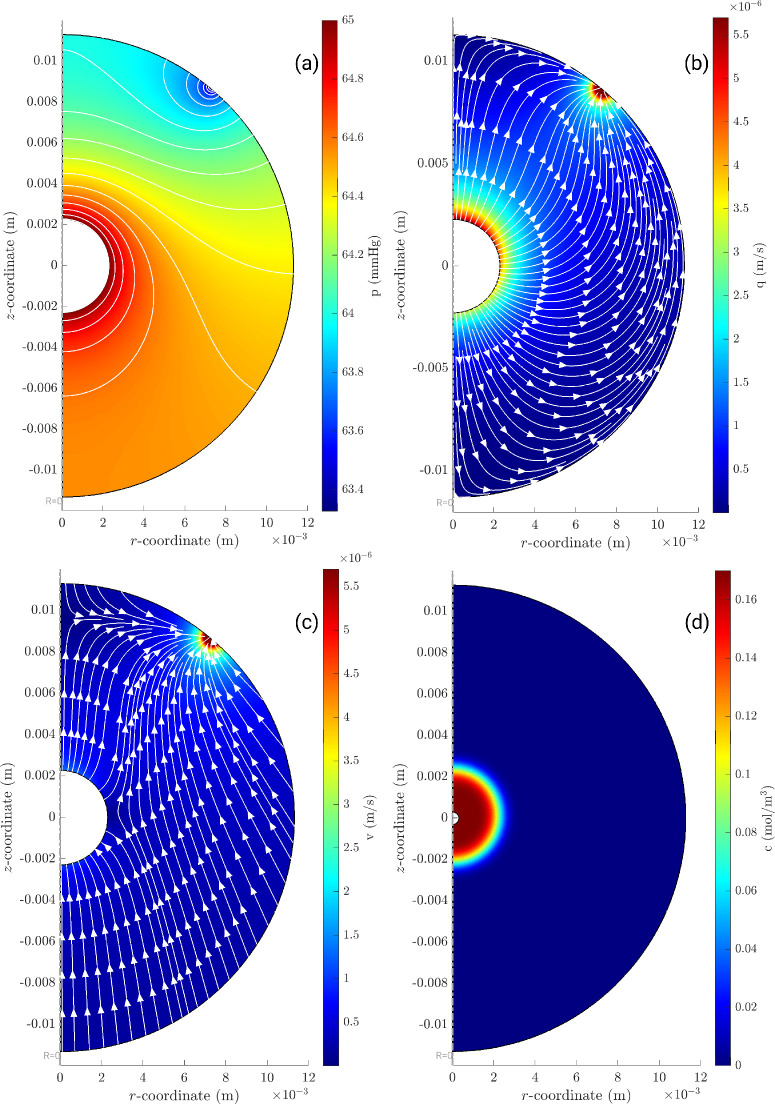
Case B, in which fluid exits through the trabecular meshwork, showing (**a**) pressure distribution *p*(***x***, 0) (mm Hg) at the initial time; (**b**) magnitude and streamlines of fluid velocity relative to the vitreous, ***q***(***x***, 0) (m/s) at the initial time; (**c**) magnitude and streamlines of fluid velocity relative to a fixed frame (max velocity 1.74 × 10^−5^ (m/s)), ***v***(***x***, 0), (m/s) at the initial time (max velocity 1.72 × 10^−5^ (m/s)); and (**d**) concentration distribution *c*(***x***, *t_fin_*) (mol/m^3^) at time *t_fin_* = 1000 seconds.

Case B is more complex. In Figure 4a, we show the pressure distribution in the domain, while Figure 4b shows the magnitude |***q***| of the fluid velocity relative to the vitreous. Both plots are at the initial time. As expected, the pressure decreases from the inner bolus (where we impose the pressure *P* computed from the lumped parameter model) to the region where the fluid exits the domain via the trabecular meshwork, $B_{s1}$. We note that pressure variations within the vitreous are small compared to *P*, which justifies our choice of imposing *P* at the inner boundary. The isobars, reported in the plot, are orthogonal to the boundary in the region $B_{s2}$, which is impermeable to fluid. Relatively strong, localized pressure gradients exist close to the outer boundary, at the region $B_{s1}$.

At subsequent times (*t* > 0), the pressure imposed at the margin of the fluid bolus, *P*(*t*), asymptotically decreases to the physiologic pressure of 15 mm Hg. At all times, the spatial distribution of the pressure remains qualitatively very similar to that shown in the figure, and the spatial pressure drop across the vitreous remains small compared to IOP.

The fluid velocity relative to the laboratory frame ***v*** is depicted by color contours in Figure 4c, overlain by the streamlines of ***v***, again at the initial time. It is interesting to notice that fluid moves from the posterior to the anterior region of the vitreous chamber and, by doing so, crosses the fluid bolus. In other words, fluid enters the inner sphere from the posterior region and exits it from its anterior side. Fluid velocity generated in the vitreous chamber at the initial time reaches a maximum value of approximately 6 µm/s at the outlet and 0.8 µm/s in the region of the injection. This flow can potentially affect the transport and distribution of the injected drug since the velocity ***v*** appears in the advection-diffusion Equation (10).

Figure 4d illustrates the computed concentration distribution of bevacizumab (along with velocity streamlines) in the domain. In this case, we show the results at the final time of our simulation (≈17 min), when the IOP has approximately returned to its baseline physiologic value and fluid flow produced during the deflation process has subsided. The bolus of drug has moved slightly toward the front of the eye (note that it is not exactly centered on the inner sphere), owing to advective effects. The boundary is also slightly blurred due to molecular diffusion.

**Figure 5.**
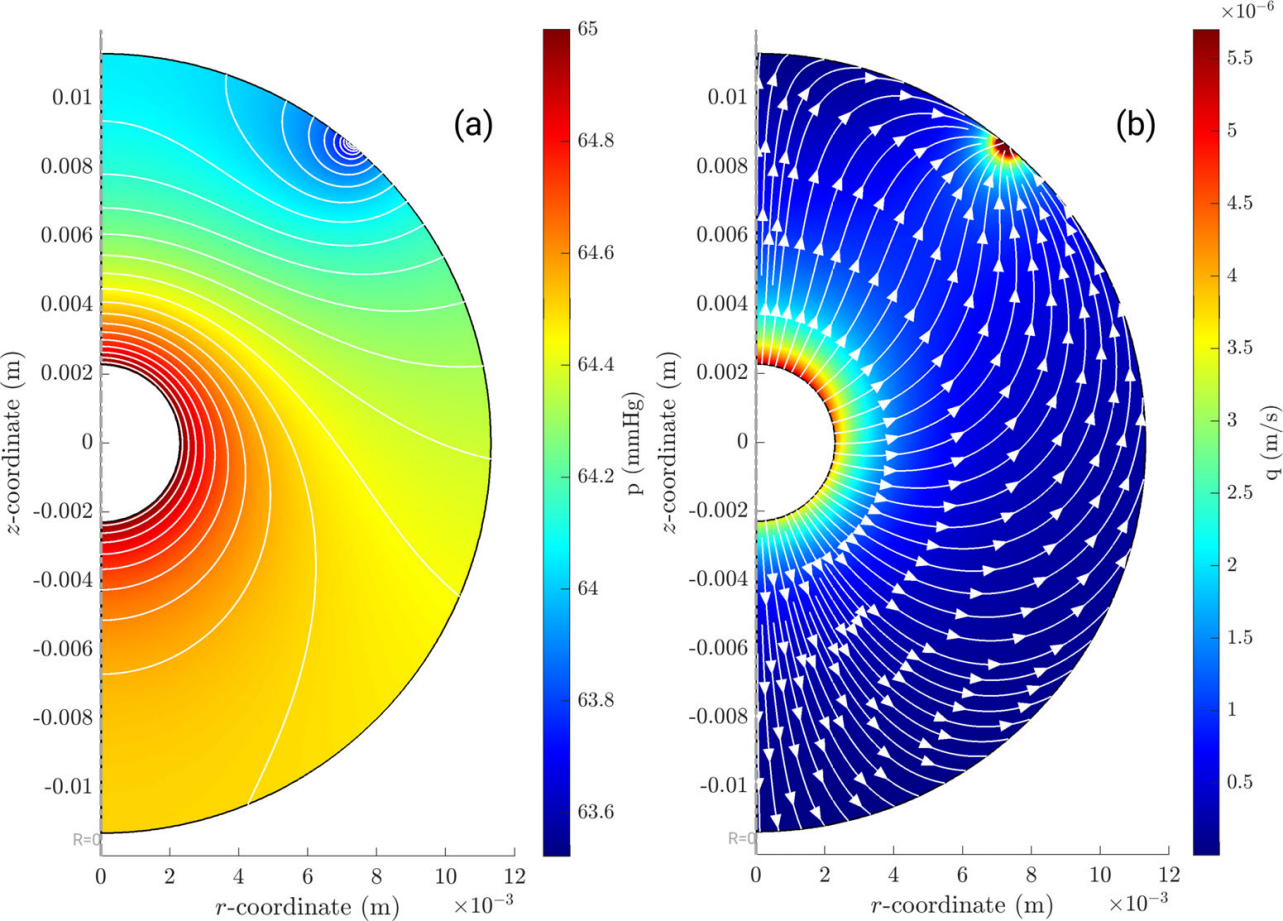
Simulation results from a case intermediate between A and B, in which 80% of fluid exits through the conventional pathway and the remainder through the sclera/unconventional pathway. (**a**) Pressure distribution *p*(***x***, 0) (mm Hg) at the initial time and (**b**) magnitude and streamlines of fluid velocity relative to the vitreous, ***q***(***x***, 0) (m/s), at the initial time (max velocity 1.39 × 10^−5^ (m/s)).

In Figure 5, we report a more realistic case, which is intermediate between case A and case B. In this case, we let 80% of the fluid to leave the domain via the conventional pathway and the remaining 20% from the unconventional pathway. Note that the streamlines of ***q*** are not orthogonal any more to the boundary of the domain since there is fluid leakage out of it. The main conclusions drawn commenting Figure 4 relative to case B also hold in this case.

## Discussion

In this work, we have presented a mechanical model to study fluid motion and drug transport within the vitreous after an intravitreal injection of bevacizumab, with the aim of assessing whether and to what extent flow associated with globe expansion and deflation may contribute to the transport of the injected drug within the vitreous.

Our model assumes that the eye is spherical and that injection creates a spherical liquid pool within the vitreous gel, which is taken to be concentric with the eye globe. We further assume that intraocular pressure and eye volume increase immediately after injection, and we predict that they progressively fall back to their original baseline values, following an exponential decay. The deflation time scale is equal to the product of ocular compliance and hydraulic resistance to aqueous outflow and is of the order of tens of minutes for a human eye.

Under the assumption that the deflation of the eye globe after injection is radially symmetric, we study fluid flow generated within the eye, assuming that the injected fluid can percolate through the vitreous gel. We consider the two following limiting cases: case A, in which fluid exits the domain uniformly through the whole scleral surface, and case B, where fluid outflow occurs only through the trabecular meshwork. Case A is representative of fluid drainage primarily via the unconventional outflow pathway (i.e., into the choroid and through the sclera). Case B models outflow via the conventional outflow pathway. In living eyes, the situation lies between these two limiting cases, 40% to 50% of aqueous humor exiting the eye through the unconventional route in nonhuman primates,^38^ although this fraction is less in humans. Unfortunately, in humans, only indirect estimates of unconventional outflow are available, which provide quite sparse data (see Table 4 of Johnson et al.^47^).

In case A (uniform fluid exit across the globe wall), the model predicts that no fluid motion is generated within the vitreous chamber during deflation, which means that the fluid flux through the sclera is entirely due to the wall motion. Thus, in this case, drug transport in the vitreous chamber only occurs by diffusion, and advection does not make any contribution. If, however, fluid can exit the domain only through a specific region (the trabecular meshwork, as in case B), fluid motion is needed to accommodate the uniform shrinkage of the eye. At the beginning of the deflation phase, this flow produces velocities of the order of some microns per second, which are significantly larger than fluid velocities generated by RPE pumping,^3^^–^^5^^,^^11^ and this motivates our interest in such a flow and its potential role on drug transport. Indeed, the Pèclet number associated with this flow, *Pe*  = *UL*/*D*, is approximately 100 at the beginning of the ocular deflation phase (here we use a characteristic velocity *U* taken close to the fluid bolus and the length scale *L* equal to the radius of the eye). This confirms the hypothesis that advection dominates drug transport at the initial times after injection. However, our results show that, overall, this flow has very little effect on drug delivery to the retina, as is clearly demonstrated by Figure 4d, which shows that advection is not capable of transporting the drug far away from the injection site. This is because the deflation phase has a duration that is too short for advection to effectively contribute to drug transport.

We note that the resistance to aqueous drainage used in the zero-dimensional model presented in the Zero-Dimensional Model of Eye Globe Deflation section is computed based on all outflow being pressure dependent, which is typically identified with the conventional pathway, since the unconventional drainage is regarded as pressure insensitive.^38^ Thus, the pressure decay computed in the Zero-Dimensional Model of Eye Globe Deflation section seems to be in contradiction with our assumptions in case A, where outflow largely occurs by uptake into the choroid and sclera (i.e., by pressure-insensitive unconventional outflow). However, Johnson et al.^47^ highlight the fact that the unconventional drainage might to some extent depend on IOP. This partially justifies our choice of considering case A. In reality, the drug transport in the eye likely lies between the predictions of cases A and B, and this situation has also been considered (see Fig. 5). The limiting cases A and B are, however, of particular interest as they cover the most extreme conditions, and in both cases, the conclusion is that fluid motion due to globe deflation does not appreciably affect drug transport.

In this context, it is of interest to mention the experimental work of Moseley et al.,^48^ in which the authors injected tritiated water into the vitreous of living rabbits and sampled vortex veins and aqueous humor in the anterior chamber to assess whether outflow occurred through the choroid or the conventional pathway. Interestingly, they found that the vast majority of injectate was recovered in the vortex veins, implying that outflow through the choroid was dominant. Of course, we note that the transport of water considered by Moseley et al.^48^ differs from the transport of a large molecule like bevacizumab, since the Pèclet number relevant to water transport is much smaller (Pe ≈ 2) than for bevacizumab transport (Pe ≈ 98), that is, diffusive transport of water is much more significant than diffusive transport of drug.

For the purposes of this work, the elastic properties of the vitreous do not play a significant role and have, therefore, been neglected. This is because the sclera is much stiffer than the vitreous gel (by several orders of magnitude), and thus the sclera is by far the structure that supports the majority of the increased pressure in the eye produced by the injection. Both the sclera and the vitreous, as is true for all tissues, have some inherent viscosity, which opposes the rate of tissue deformation. However, the viscous contribution that resists eye deflation is essentially due to the resistance to fluid outflow, which dwarfs the effect of scleral and vitreous viscosities. This justifies neglecting viscosity of the sclera and the vitreous.

The vitreous body has a complex microstructure and is not isotropic. Accounting for anisotropy of vitreous permeability in the model would not lead to conceptual complications, that is, the scalar permeability *k* in Equation (8) would be replaced by a permeability tensor. This, however, would require significant speculation about the values of this tensor for which experimental data are sparse or entirely lacking. Moreover, our simulations show the advective drug transport, which is related to water flow, is largely irrelevant, and this conclusion would not be modified by inclusion of the anisotropy of vitreous permeability.

Vitreous structure and its anisotropy could in principle also have a role in the diffusion processes. However, as we have noted above, the diffusion of bevacizumab within the vitreous is likely to be unhindered (i.e., essentially the same as would be expected for diffusion of bevacizumab in aqueous solution). This is justified on the basis of the high porosity of the vitreous and is also generally consistent with the work of Zhang et al.^49^ These authors experimentally measured the diffusion of bevacizumab in the rabbit vitreous, obtaining a fitted diffusivity of *D* = 12   ±   6 × 10^−11^ m^2^s^−1^, slightly higher than our value of *D* = 7  × 10^−11^ m^2^s^−1^. Zhang et al.^49^ did not account for convection in their data-fitting process, and thus their diffusivity may be a slight overestimate of *D*. In any case, it appears that the value of *D* that we utilized is reasonable.

The vitreous gel typically degrades with age, and this can lead to the formation of liquid pools in the vitreous cavity. The main conclusion of this work—namely, that fluid motion generated during deflation of the eye after an intravitreal injection contributes negligibly to drug transport—is expected to apply also to the case of a liquefied vitreous. However, when the vitreous gel is extensively liquefied, drug transport in the vitreous chamber is expected to be dominated by fluid motion generated by eye rotations.^9^^,^^12^^–^^14^

We have considered a highly idealized geometry and modeled the eye as a perfect sphere. Although this is a somewhat crude approximation, it is a reasonable first step, and we do not expect that our conclusions would be markedly different if a more realistic geometry was considered. This is because pressure variations within the domain, which obviously depend on the domain shape, are found to be very small compared to the overall IOP variations. Moreover, within the time scale of interest, the injected drug is predicted to remain close to the injection site, and therefore, the distribution of drug concentration is only very modestly affected by the shape of the boundary.

In conclusion, despite the relatively large velocities that are generated during the eye globe deflation after an intravitreal injection, this fluid motion does not significantly contribute to drug transport in the eye and can be safely neglected.

**Table.** Parameter Values

Parameters and Data	Symbols	Value	Species	Reference
Angle subtended by aqueous drainage structures on corneoscleral shell	Δθ	2 deg	—	Derived
Aqueous humor production rate minus pressure-independent outflow rate	*Q_in_*	2.2 µL/min	Human	^40^
Aqueous outflow resistance	R	3.18 mm Hg min/µL	—	Derived
Diffusion coefficient of bevacizumab	*D*	7 × 10^−11^ m^2^/s	—	Derived
Bevacizumab concentration	*c*_0_	0.17 mol/m^3^	Human	^50^
Episcleral venous pressure	*P_epv_*	8 mm Hg	Human	^28^ ^–^ ^30^
Injected volume of drug solution	*V_inj_*	50 µL	Human	^51^
Intraocular pressure in physiologic conditions	$\hat{P}$	15 mm Hg	Human	^18^ ^–^ ^20^
Ocular compliance	C	1 µL/mm Hg	Human	^21^
Ocular volume in physiologic conditions	$\hat{V}$	6000 µL	Human	^52^
Vitreous hydraulic conductivity	*k*/μ	8.4 × 10^−7^cm^2^/(Pa s)	Bovine	^5^
